# Incidence of prostate cancer in transgender women in the US: a large database analysis

**DOI:** 10.1038/s41391-024-00804-4

**Published:** 2024-02-07

**Authors:** Matthew Loria, David Gilbert, Tomasz Tabernacki, Mart Andrew Maravillas, Megan McNamara, Shubham Gupta, Kirtishri Mishra

**Affiliations:** 1 University Hospitals, Urology Institute, Cleveland, OH USA; 2 Case Western Reserve University School of Medicine, Cleveland, OH USA; 3 Louis Stokes Veterans Association Cleveland, Cleveland, OH USA; 4 Metro Health Hospitals, Cleveland, OH USA

**Keywords:** Cancer epidemiology, Cancer epidemiology, Prostate cancer

## Abstract

The risk of prostate cancer among transgender women undergoing medical and surgical gender-affirming interventions remains unclear, though up to a fivefold decreased risk has been reported in comparison to cisgender men. In this study, we conducted a comparative analysis of the risk of prostate cancer among transgender women (TW) using data from TriNetX, a large database, versus SEER. Our findings indicate that, overall, transgender women exhibited a 2.56-fold lower risk of prostate cancer compared to cisgender men. Specifically, among TW on hormone therapy between ages 50–64, we observed a 2.06-fold decrease in risk. Contrary to the previous perception of prostate cancer being rare in transgender women, our study suggests that it may not be as uncommon as previously believed.

## Introduction

After transgender women (TW) undergo genital gender-affirming surgery the prostate remains in situ conferring an ongoing risk for prostate cancer (PCa). However, the incidence of prostate cancer in this population is relatively unknown [1]. Current evidence-based guidelines for PCa screening in TW are limited by this minimal data on PCa incidence. When compared to the rates of PCa in cisgender men, some studies report between a two to fivefold decrease in the risk of PCa [2, 3]. This study aims to investigate the rates of PCa in TW who have undergone medical or surgical transition to better inform screening guidelines for this population.

## Methods

We used the TriNetX database to access deidentified ehealth records for 120 million patients and over 40,000 TW from the United States. This data was used to perform a retrospective cohort study comparing the rates of PCa among TW. TW were identified using ICD-10 code F64 which has been shown to be proficient in identifying transgender individuals (See Supplementary 1). Three cohorts were created using ICD-10 codes: TW with no medical or surgical intervention (NI), TW with estrogen hormone therapy but no surgical intervention (HT), and TW with HRT and gender affirming surgery, defined specifically by having an orchiectomy (SX) (See Supplementary 1). Patients were excluded if they had a diagnosis of PCa prior to their index event. Additionally, to reduce the likelihood of inclusion of individuals in the SX cohort who underwent orchiectomy for reasons other than gender affirmation, those with an orchiectomy prior to hormone therapy were excluded.

To assess the incidence rates of prostate cancer among transgender women in comparison to the general population, a standardized incidence ratio (SIR) was calculated. Prostate cancer incidence rates were derived from the National Institutes of Health Surveillance, Epidemiology, and End Results (SEER) program, specifically utilizing 5-year age-adjusted data spanning from 2016 to 2020. These rates served as the basis for estimating the expected cases within our study population. A thorough comparison between the observed cases, from TriNetX, and the expected cases, as determined by the NIH SEER data, enabled the calculation of the standardized incidence ratio. Further comprehensive details, including baseline characteristics, can be found in the electronic Methods section provided in Supplementary 1.

## Results

In our cohort of 40,727 TW, we observed 43 cases of PCa (Table 1) during an average 3 year follow up. We found that TW on HT between 50–64 had a prostate cancer risk of 0.48 (Table 1), while TW on HT of all ages had a risk of 0.22 compared to SEER data. The HT group was on hormone therapy for an average of 2 years prior to PCa diagnosis (Table 2).

**Table 1:** Prostate cancer standardized incidence ratio by cohort.

Cohort (age at index)	Follow up time (years)	Observed cases	Expected	US incidence rate (per 100,000 person-years)	Standardized incidence ratio (95% Confidence interval)
No Intervention (NI)					
<50	35,284	4	1	3.6	3.15 (0.82–6.99)
50–64	3679	13	9	235.9	1.50 (0.79–2.42)
65+	1078	11	6	596.7	1.71 (0.85–2.87)
Overall	40,041	28	45	113.1	0.62 (0.41–0.87)^a^
Hormone Therapy (HT)					
<50	48,818	3	2	3.6	1.71(0.31–4.19)
50–64	5346	6	13	235.9	0.48 (0.17–0.93)^a^
65+	1266	5	8	596.7	0.66 (0.21–1.37)
Overall	55,430	14	63	113.1	0.22 (0.12–0.36)^a^
HT + Surgical Intervention (SX)					
<50	1154	1	0	3.6	24.07 (0.01–94.37)
50–64	61	0	–	235.9	–
65+	6	0	–	596.7	–
Overall	1,221	1	1	113.1	0.72 (0.00–2.84)
All Trans Women					
<50	85,256	8	3	3.6	2.61 (1.11–4.73)^a^
50–64	9086	19	21	235.9	0.89 (0.53–1.33)
65+	2350	16	14	596.7	1.14 (0.65–1.77)
Overall	96,692	43	109	113.1	0.39 (0.28–0.52)^a^

^a^Significant.

**Table 2:** Characteristics of transgender women who were diagnosed with prostate cancer.

	No intervention (NI) *n* = 28	Hormone therapy (HT) *n* = 14	HT + Surgical intervention (SX) *n* = 1	Overall *n* = 43
PSA at the time of diagnosis in ng/mL (Median, Range)	5.2, 0.05–31.12	17.18, 0.4–841.81	(NA)	6.24, 0.05–841.41
Age at the time of diagnosis in years (Median, Range)	64.5, 24–88	64.5, 31–85	(38)	63, 24–88
Amount of time when a patient was diagnosed after index event years (Median, Range)	NA	2, <1–6	(<1)	2, <1–6

() – sample size < 2.

In the NI group, we had a decreased PCa risk compared to cis-men (0.62) (Table 1). However, when age stratified, the risk of PCa was not significant across all age groups (Table 1). Among TW who were both on HT and had surgery, only 1 case of PCa was observed in a patient 38 years of age. Of all those diagnosed with PCa, the median PSA among HT at time of diagnosis was 17.18. See supplementary results for more data.

## Discussion

In our study, which represents the largest cohort of transgender women (TW) examined for prostate cancer (PCa) to date, we found a twofold lower incidence of PCa among trans women between ages 50–64 receiving hormone therapy (HT). Additionally, when considering the incidence among all TW in our study, we identified a 2.56-fold lower incidence of PCa. This may, in part, be attributed to the overall younger age of the TW population. However, even when age-stratified and accounting for follow-up time, we observed a 2-fold lower incidence of PCa among TW on HT between ages 50–64.

Previous literature has consistently established that PCa is less common among TW, however the extent of this reduction has not been well-defined [4]. A significant contribution to the field comes from the Netherlands by Di Nie et al., where a retrospective cohort study of 2281 trans women demonstrated a fivefold lower incidence of prostate cancer among those on HT [2]. Notably, less research has explored PCa rates in the US population. A recent case series by Nik-ahd et al. indicated a twofold lower incidence of PCa in a cohort of 449 transgender women veterans on HT [3]. Therefore, our extensive database analysis, comprising 40,000 TW, builds upon the work of Nik-ahd et al. and challenges the prevailing belief that PCa is exceedingly rare in the United States TW population.

Furthermore, underlying reasons for the lower PCa rates observed may include factors such as reduced PSA screening, the suppressive effects of estrogen on PCa development, or the possibility of misdiagnosis due to misinterpretation of PSA levels in individuals undergoing gender-affirming hormone treatment [5].

Among those diagnosed with PCa in our study, the average prostate-specific antigen (PSA) was 17.18 ng/mL (Range: 0.4–841.41). Elevated PSAs have been linked to advanced staging and increased aggressiveness of PCa [6]. This suggests that the disease burden of TW diagnosed with PCa may be higher, posing potential challenges in therapy. However, the interpretation of increased PSA in this context is limited until further research establishes normal PSA ranges in individuals undergoing gender affirming hormone therapy [7].

Acknowledging the limitations inherent in large electronic medical record (EMR) database studies, including potential issues related to the completeness and accuracy of medical records, we also recognize that the young average age of our cohort may impact the generalizability of our findings, as prostate cancer becomes more prevalent with age. We attempted to address this through indirect age-adjustment. Additionally, the age distribution of our cohort closely mirrors that of transgender individuals in the United States [8]. However, this may limit research on diseases of older age in the trans community, which will gain significance as this population ages.

In conclusion, our study reports a twofold decrease in the incidence of prostate cancer in TW on HT between 50 and 64 years old. Contrary to previous assumptions, this study suggests that PCa may not be as exceedingly rare among TW as once believed. While rates are still decreased relative to cisgender men, occurrences are not infrequent, necessitating healthcare providers consider PCa when managing the health of TW.

For future studies, efforts should prioritize investigating the causative factors behind these reduced PCa rates in transgender women to guide clinical practice effectively. Further research should work to enhance strategies for detecting prostate cancer in transgender women.

## Supplementary information

Supplemental Methods

## Data Availability

The data that support the findings of this study are available from TriNetX but restrictions apply to the availability of these data, which were used under license for the current study, and so are not publicly available. Data are however available from the authors upon reasonable request and with permission of TriNetX.
