# Supplementary material for: Incidence of prostate cancer in transgender women in the US: a large database analysis
Source: Prostate Cancer Prostatic Dis. 2024 Feb 7;28(1):232–4. doi: 10.1038/s41391-024-00804-4 (PMC11860230; doi:10.1038/s41391-024-00804-4)
Supplement: Supplementary file 1 — Supplemental Methods [file 41391_2024_804_MOESM1_ESM.docx]

**Supplemental**

**eMethods**

The TriNetX database contains data from over 120 million patients who are enrolled in 100+ healthcare organizations across the United States and North America. 72 of these healthcare organizations participated in our query. Data was relatively equally distributed across the United States ( 30% West, 23% Northeast, 24% Midwest and 22% South). All clinical data is de-identified and comes directly from electronic medical records (EMR) of HCOs. University Hospitals institutional review board determined that data from TriNetX is not human subject research and is therefore exempt from approval. TriNetX collects data from all types of insurance and HCOs.

TriNetX completes an intensive data preprocessing stage to minimize missing values. Data are mapped to a standard and controlled set of clinical terminologies and converted to a proprietary data schema. This transformation process includes an extensive data quality assessment to reject records that do not meet quality standards. Quality assurance of the data is performed using a standardized format before integration into the database.

All covariates are either binary, categorical (which expands to a set of binary columns), or continuous but essentially guaranteed to exist. Age is guaranteed to exist. Missing sex values are represented using “Unknown Sex”. The missing data for race and ethnicity are presented as “Unknown race” or “Unknown Ethnicity”. For other variables including medical conditions, procedures, lab tests, and socio-economic determinant health, the value is either present or absent so “missing” is not pertinent.

Data was downloaded and analyzed by biostatisticians affiliated with University Hospitals. Nearly all patient data for TW ranged from 2016-2023, but data from TriNetX ranges from 2000 to 2023. Patients over the age of 18 with a F64 Gender Identity Disorder ICD-10 code were divided into cohorts based on treatment received per ICD-10 codes. Age at the index event was reported.

Cohorts were established using the EMR codes below. NI cohort inclusion criteria were GID category and exclusion was HRT and Surgical intervention. NI Cohort index event was the date of GID diagnosis. HT cohort inclusion criteria were GID and HRT categories and exclusion was surgical intervention category. HT index event was the first date on which GID and HRT codes were present in the patients’ medical record. SX cohort had all three categories as inclusion; their index event is the first day all three categories were achieved. Orchiectomy ICD-10 codes were chosen for SX group and physiological differences were examined. However, this decision is limited by coding nuances which may result in under-representation of TW who have had gentital gender affirming surgery. Several studies have demonstrated that ICD-10 codes represent the best method to identify transgender individuals in electronic health records and have a high specificity but an uncertain sensitivity [1-3]. ICD code identification of transgender status ranged from 88-100% [4]. Prostate Cancer was assessed with ICD 10 code C61 and based upon the date of entry into EHR.

Additional data including PSA, age, and time since the index event was analyzed at diagnosis date. Baseline characteristics were assessed for differences using Kruskal-Wallis rank sum test, Pearson’s chi-square test; and fisher’s exact test. Prostate cancer rates were determined using Standardized Incidence Ratio along with 95% confidence intervals. Rates in TriNetX were compared to SEER 5-Year Age-Adjusted Incidence Rates from 2016-2020.

Table 1

| **Criteria Category** | **Description** | **Code** | **Code Type** |
| --- | --- | --- | --- |
| **Gender Identity Disorder (GID)** | Transsexualism | F64.0 | ICD-10-CM |
|  | Dual role transvestism | F64.1 | ICD-10-CM |
|  | Gender Identity Disorder of Childhood | F64.2 | ICD-10-CM |
|  | Other gender identity disorders | F64.8 | ICD-10-CM |
|  | Gender identity disorder unspecified | F64.9 | ICD-10-CM |
| **HRT** | ESTROGENS | G03C | ATC |
|  | Estrogens | L02AA | ATC |
|  | ESTROGENS | HS300 | VA |
|  | Hormone Replacement Therapy | Z79.890 | ICD-10-CM |
| **Surgical Intervention** | Orchiectomy, simple (including subcapsular), with or without testicular prosthesis, scrotal or inguinal approach | 54520 | CPT |
|  | Laparoscopy, surgical; orchiectomy | 54690 | CPT |

**Figure 1. Inclusion Criteria**


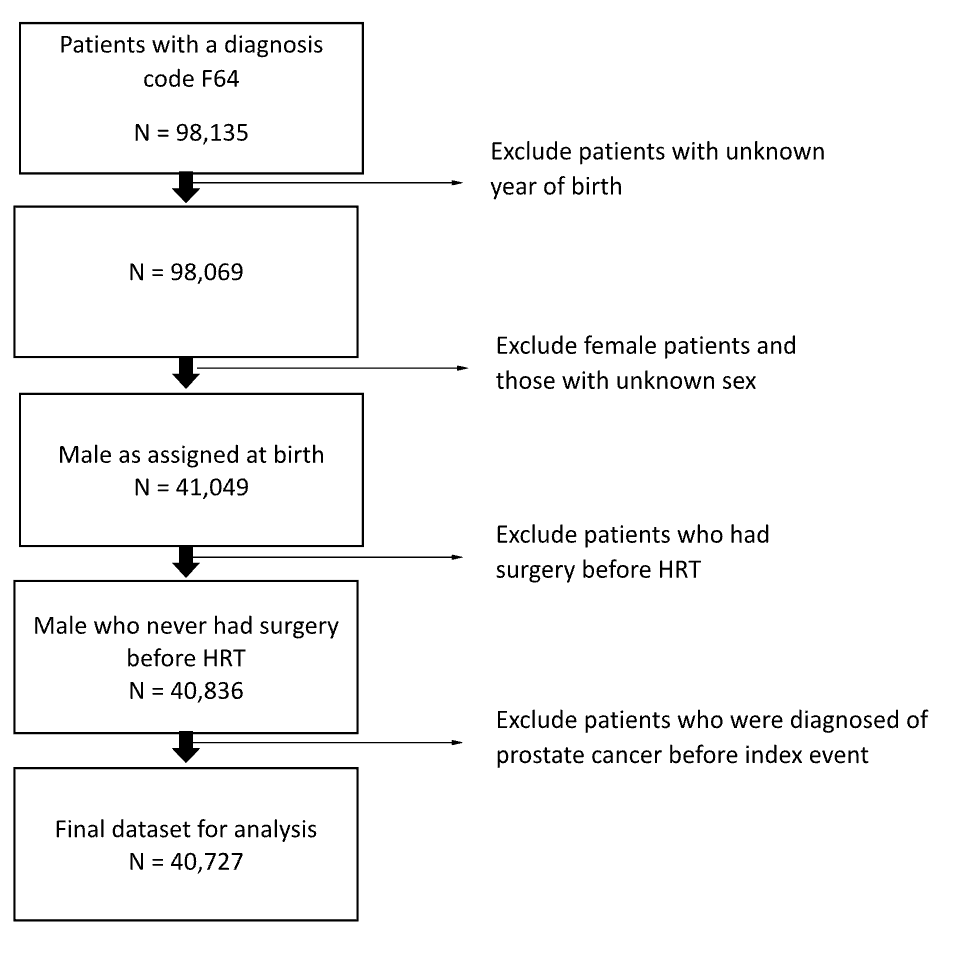


**Supplemental Results**

**Table 2. Baseline characteristics of the study cohort.**

| **Characteristic** | **Overall**  **n = 40,727** | **No Intervention (NI)**  **n = 19,742** | **Hormone Therapy (HT)**  **n = 20,448** | **HT + Surgical Intervention (SX)**  **n = 537** | **p-value** |
| --- | --- | --- | --- | --- | --- |
| **Age at index** (mean ± SD) | 28.23 ± 13.98 | 27.8 ± 14.9 | 28.45 ± 12.98 | 36.1 ± 13.29 | <0.001^a^ |
| **BMI** (mean ± SD) | 26.19 ± 7.04 | 25.83 ± 7.25 | 26.26 ± 6.91 | 27.77 ± 6.97 | <0.001^a^ |
| **Race (n, %)** |  |  |  |  | <0.001^b^ |
| Black | 3,714 (9.12%) | 1,940 (9.83%) | 1,757 (8.59%) | 17 (3.17%) |  |
| White | 27,675 (67.95%) | 12,804 (64.86%) | 14,466 (70.75%) | 405 (75.42%) |  |
| Other^c^ | 1,456 (3.58%) | 792 (4.01%) | 644 (3.15%) | 20 (3.72%) |  |
| Unknown | 7,882 (19.35%) | 4,206 (21.3%) | 3,581 (17.51%) | 95 (17.69%) |  |
| **Ethnicity (n, %)** |  |  |  |  |  |
| Hispanic or Latino | 3,240 (7.96%) | 1,575 (7.98%) | 1,645 (8.04%) | 20 (3.72%) | <0.001^b^ |
| Not Hispanic or Latino | 27,752 (68.14%) | 12,722 (64.44%) | 14,638 (71.59%) | 392 (73%) |  |
| Unknown | 9,735 (23.9%) | 5,445 (27.58%) | 4,165 (20.37%) | 125 (23.28%) |  |
| **Follow-up time in years** (mean ± SD) | 2.37 ± 2.86 | 2.02 ± 2.88 | 2.74 ± 2.82 | 1.59 ± 1.71 | <0.001^a^ |
| **Prostate cancer incidence** | 43 (0.11%) | 28 (0.14%) | 14 (0.07%) | ≤10 (0.19 – 1.8%) | 0.041^b^ |
| ^a^Kruskal-Wallis rank sum test  ^b^Pearson’s chi-squared test; Fisher’s exact test  ^c^Other includes Asian, Native American, and Native Hawaiian or Other Pacific Islander | | | | | |

**Supplemental Methods References**

1. Dubin, S., Cook, T., Liss, A., Doty, G., Moore, K., Greene, R., ... & Janssen, A. (2022). Comparing Electronic Health Record Domains' Utility to Identify Transgender Patients. Transgender Health, 7(1), 78-84.
2. Jasuja, G. K., de Groot, A., Quinn, E. K., Ameli, O., Hughto, J. M., Dunbar, M., ... & Rose, A. J. (2020). Beyond gender identity disorder diagnosis codes: An examination of additional methods to identify transgender individuals in administrative databases. Medical care, 58(10), 903.
3. Roblin D, Barzilay J, Tolsma D, et al. A novel method for estimating transgender status using electronic medical records. Ann Epidemiol. 2016;26:198–203.
4. Nik-Ahd, F., Waller, J., De Hoedt, A. M., Garcia, M. M., Figueiredo, J. C., Carroll, P. R., ... & Freedland, S. J. (2023). Seeing the unseen: how can we best identify transgender women within the Veterans Affairs healthcare system’s electronic medical record?. The Journal of Sexual Medicine, 20(4), 559-567.
